# Influence of particle size in supercritical carbon dioxide extraction of roselle (*Hibiscus sabdariffa*) on bioactive compound recovery, extraction rate, diffusivity, and solubility

**DOI:** 10.1038/s41598-023-32181-8

**Published:** 2023-07-05

**Authors:** Nicky Rahmana Putra, Dwila Nur Rizkiyah, Ahmad Hazim Abdul Aziz, Hasmadi Mamat, Wan Muhammad Syahir Wan Jusoh, Zuhaili Idham, Mohd Azizi Che Yunus, Irianto Irianto

**Affiliations:** 1 Centre of Lipid Engineering and Applied Research (CLEAR), Ibnu Sina Institute for Scientific and Industrial Research, Universiti Teknologi Malaysia, UTM Johor Bahru Campus, 81310 Johor Bahru, Malaysia; 2 Faculty of Food Science and Nutrition, Universiti Malaysia Sabah, 88400 Kota Kinabalu, Malaysia; 3 Department General Education, Faculty of Resilience, Rabdan Academy, Abu Dhabi, United Arab Emirates

**Keywords:** Chemical engineering, Engineering

## Abstract

The purpose of this work was to establish the best particle size for recovering high yields of total phenolic compounds (TPC), total anthocyanin compounds(TAC) and total flavonoid compounds (TFC) from roselle (*Hibiscus sabdariffa*) by applying supercritical carbon dioxide (ScCO_2_). The extraction rate, diffusivity and solubility of yield in ScCO_2_ were also studied and calculated utilizing models. Pressure (10 and 30 MPa), temperature (40 and 60 °C), and particle size (250 µm < dp < 355 µm, 355 µm < dp < 425 µm and 425 µm < dp < 500 µm) were employed as variables in this experiment. The greatest recovery was 11.96% yield, 7.16 mg/100 g TAC, 42.93 mg/100 g TPC and 239.36 mg/100 g TFC under the conditions of 30 MPA, 40 °C and 250 µm < dp < 355 µm, respectively. The extraction rate of supercritical carbon dioxide in roselle extraction ranged from 5.19 E−03 to 1.35 E−03 mg/s fitted using the Esquivel model. The diffusivity coefficient of ScCO_2_ ranged from 2.17E−12 to 3.72E−11 mg/s^2^, as fitted by a single sphere model. The greatest solubility of global yield, TAC, TPC and TFC in ScCO_2_ was 1.50 g/L, 0.3 mg/L, 1.69 mg/L and 9.97 mg/L, respectively, with a particle size of 250 µm < dp < 355 µm. The smaller particle size of roselle provides the maximum bioactive compound recovery and solubility. Furthermore, the diffusivity and extraction of ScCO_2_ are increased by decreasing the particle size. Therefore, a smaller particle size is appropriate for roselle extraction by ScCO_2_ based on the experimental and modelling data.

## Introduction

Roselle (*Hibiscus sabdariffa* L.) has a lengthy history of usage in a range of medical fields. It is used to treat liver damage, hypertension, and leukemia, among other disorders. Additionally, it has a number of medicinal benefits that have been researched internationally^1^. Apart from being offered in the form of drinks such as jellies, fruit juices and dried fruit, it is also exploited as a colouring factor in a number of sectors^2^. Additionally, roselle contains a high concentration of phenolic, flavonoid, and antioxidant components^3^.

Current research demonstrates that supercritical carbon dioxide (ScCO_2_) is often utilized to extract important chemicals, notably phenolic, flavonoid and antioxidant compounds. ScCO_2_ is an innovative approach to optimize the extraction of phenolic compounds because this solvent is safe and green. The key advantages of this technique over conventional extraction include reduced extraction time, increased extract quality, lower extraction agent costs, and an environmentally friendly method^4^. This method is fast gaining acceptance as a feasible alternative to solvent-solid extraction^5^. Its usage in the domain of essences is very recent and extremely promising^6^. Previous study shows that this method is applied to extract *Cleome coluteoides Boiss*^7^*, L. acanthodes*^8^*,* peanut skin^9^, omega-3 from Dracocephalum kotschyi seed oil^10^ and rosmarinic acid from *Orthosiphon stamineus*^11^.

There are two factors, chemicals and mechanical factors, in supercritical carbon dioxide extraction^12,13^. Researchers commonly use chemical factors such as pressure and temperature to enhance bioactive compound recovery. On the other hand, mechanical factors, such as the particle size of materials, have been slightly highlighted by researchers to enhance the extraction process^7,14^. The particle size of roselle was studied to determine the best particle size to obtain high global yield and anthocyanin, phenolic and flavonoid compounds.

The modeling is utilized to evaluate the particle size data and assess the effect of particle size on the extraction process. There are two models, Brunner and Esquivel, used to calculate the extraction rate of ScCO_2_ affected by particle size as a factor^15,16^. Furthermore, the diffusivity of ScCO_2_ impacted by particle size is explored and calculated using single sphere models^17^. The solubility of global yield, anthocyanin, phenolic and flavonoid chemicals impacted by particle size is also determined. Therefore, this study is complete in terms of experimental analysis and supported by modeling analysis to determine the influence of particle size to enhance supercritical carbon dioxide extraction.

Therefore, there are two objectives in this research. First, the focus of this research was to define the best particle size for recovering high yields, total anthocyanin compounds (TAC), total phenolic compounds (TPC) and total flavonoid compounds (TFC) from roselle by employing ScCO_2_. The second objective was to study the extraction rate, diffusivity and solubility of yield in ScCO_2_ influenced by particle size utilizing a model.

## Materials and methods

### Preparation of roselle

The supplier of the dried roselle (*Hibiscus sabdariffa)* was Ekomekar Resources in Terengganu and grounded to various particle sizes of 250 µm < dp < 355 µm, 355 µm < dp < 425 µm and 425 µm < dp < 500 µm using a professional blender (Panasonic, Japan) and sieved using an Endecott’s Octagon 2000 Digital Sieve Shaker. The origin of the roselle is from the Terengganu, Malaysia. The moisture content of dried roselle was maintained below than 8%, that the moisture content (%) was calculated from the sample weight before and after drying. The dried was in a freezer (Liebherr EFL 3505).

### Chemicals

Sigma-Aldrich provided ethanol analysis grade (99.50%), gallic acid, Folin-Ciocalteu, KCl and Na_2_SO_4_. Anhydrous of Na_2_CO_3_. Al_2_NO_3_ and CH_3_COOK were also purchased from Sigma-Aldrich. Liquid CO_2_ (99% purity) was used in the solid-solvent extraction purchased from Kras, Johor Bahru, Malaysia.

### Supercritical carbon dioxide (ScCO_2_)

**Figure 1:**
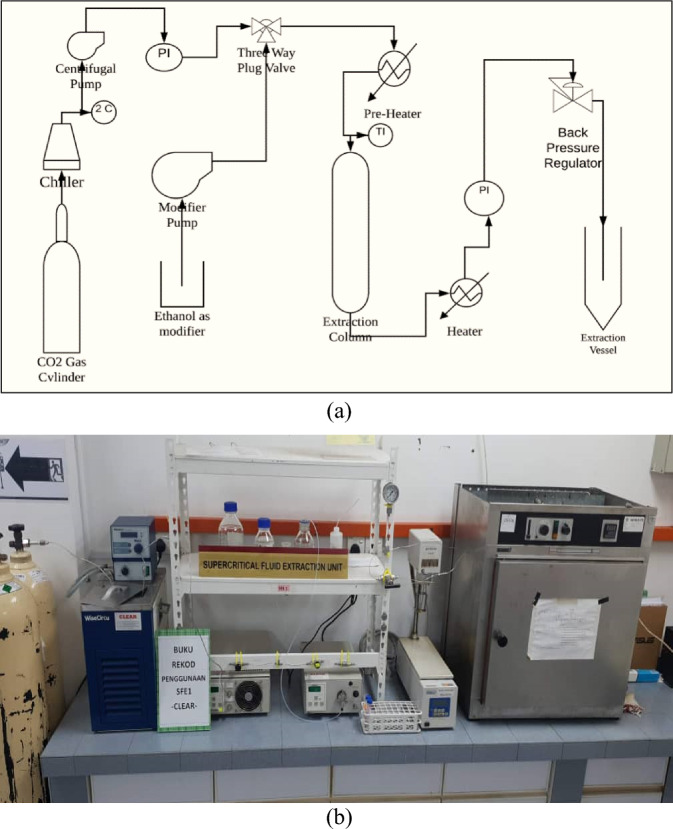
The ScCO_2_ extraction (**a**) schematic diagram (**b**) apparatus.

The equipment consisted of a 50 mL extraction vessel (internal diameter: 1.4 cm; length: 33 cm), a CO_2_ pump (Lab Alliance's Supercritical 24), a back pressure regulator (Jasco BP 2080 Plus Automated BPR, Japan), and an oven (Memmert, Japan). Roselle powder (3 ± 0.005 g) was added to an extraction vessel, and the CO_2_ temperature chiller was adjusted to 6 °C. To find the optimal particle size, particle sizes of 250 µm < dp < 355 µm, 355 µm < dp < 425 µm and 425 µm < dp < 500 µm were used. The back-pressure regulator's heater was set to 50 °C. After slowly adding liquid CO_2_, 0.24 mL/min ethanol was introduced as an entrainer (V_Etoh_/V_CO2_). The extraction time was set to 60 min. The extract contained the ethanol as a co-solvent was dried using vacuum evaporator at temperature of 40 °C to prevent the degradation process. Table 1 summarizes the extraction parameters and responses. The schematic design of ScCO_2_ extraction utilizing ethanol as a cosolvent is shown in Fig. 1.

**Table 1:** The particle size parameters and responses of yield (%), TAC (mg/100 g), TPC (mg/100 g) and TFC (mg/100 g).

Run	Temp, °C	P, Mpa	F_CO2_, ml/min	F_Modifier_, ml/min	Time, min	Dp, µm	Global yield, %	TAC, mg/100 g	TPC, mg/100 g	TFC, mg/100 g
1	40	10	4	0.24	60	250 < dp < 355	7.91	3.12	25.98	130.27
2						355 < dp < 425	6.91	2.09	21.28	123.27
3						425 < dp < 500	6.37	1.50	15.08	101.81
4	60	10	4	0.24	60	250 < dp < 355	7.93	2.59	22.37	120
5						355 < dp < 425	7.75	2.04	19.66	105.44
6						425 < dp < 500	6.78	1.70	15.76	93.63
7	40	30	4	0.24	60	250 < dp < 355	11.96	7.16	42.93	239.36
8						355 < dp < 425	11.57	6.28	39.32	219.27
9						425 < dp < 500	9.15	5.71	34.06	191.81
10	60	30	4	0.24	60	250 < dp < 355	8.99	6.66	39.67	231
11						355 < dp < 425	8.43	5.35	35.93	200.90
12						425 < dp < 500	6.59	4.82	31.86	170.90
Average							8.36	4.09	28.66	160.64

### Analysis of total anthocyanin content (TAC)

A pH differential technique was used to assess the anthocyanin content of roselle calyces extract^18^. Two dilutions of the same material were prepared using potassium chloride (0.025 M) and sodium acetate trihydrate solutions (0.4 M), respectively. Both were adjusted to pH 1.0 and 4.5, respectively, using hydrochloric acid (0.1 M). The absorbance was determined using an ultraviolet–visible (UV–Vis) spectrophotometer (Jasco, Japan) at 520 and 700 nm using Eq. (1).

1 $${\text{Absorbance}}: \, \left( {{\text{A}}_{{{52}0}} - {\text{ A7}}_{00} } \right){\text{pH}}_{{{1}.0}} {-} \, \left( {{\text{A}}_{{{52}0}} - {\text{ A}}_{{{7}00}} } \right)_{{{\text{pH 4}}.{5}}} .$$

The TAC was calculated as mg cyanidin-3-glucoside (cya 3-glu)/100 g of dry roselle as in Eq. (1);

2 $${\text{TAC }}\left( {{\text{mg}}/{\text{L}}} \right):{\text{ A }} \times {\text{ MW }} \times {\text{ DF }} \times { 1}000/\varepsilon \, \times {\text{ L}}{.}$$

A is absorbance, MW is the molecular weight of cyanidin 3-glucoside (449.2 g/mol), DF is the dilution factor, Ɛ is the cyanidin 3-glucoside extinction coefficient (26,900 L/cm mol), and L is the cell path length (1 cm). TAC (mg/L) was transformed to mg of roselle extract per 100 g of dried roselle.

### Analysis of total phenolic compounds (TPC)

According to Rizkiyah et al.^3^, the total phenolic compounds in each sample were determined. 5 mL of Folin–Ciocalteu reagent was sonicated for 5 min in 50 mL of distilled water and 40 mL of distilled water containing 3 g of Na_2_CO_3_. The concentration of extract was set 1 mg/mL. Furthermore, 1 mL of diluted extract was added 5 mL of Folin–Ciocalteu solution. The addition of 4 mL of Na_2_CO_3_ solution was followed by a 30 min rest at room temperature. Using a UV–Vis spectrophotometer, the absorbance at 760 nm was measured (Jasco, Japan). The limit of detection (LOD) and limit of quantification (LOQ) were 23.31 and 70.63, respectively. The total phenolic compounds were analyzed using a gallic acid standard curve, and the results are reported in milligrams of gallic acid equivalents per 100 g of dried roselle (mg/100 g).

### Analysis of total flavonoid compounds (TFC)

The analysis of total flavonoid compounds was conducted using Putra et al.^19^. The concentration of extract was set 1 mg/mL. 1 mL of sample was mixed with 0.2 mL of 10% Al_2_NO_3_ and 0.2 mL of 1.0 M CH_3_COOK at room temperature for 40 min. At 415 nm, the absorbance of each sample was measured using a UV–Vis spectrophotometer (Jasco, Japan). The limit of detection (LOD) and limit of quantification (LOQ) were 21.34 and 64.67, respectively Using quercetin as a standard, a standard curve was constructed, and the results were reported as mg quercetin equivalents per 100 g of dried roselle (mg/100 g).

### Experimental design

Pressure (10 and 30 MPa), temperature (40 and 60 °C), and particle size (250 µm < dp < 355 µm, 355 µm < dp < 425 µm and 425 µm < dp < 500 µm) were employed as variables in this experiment. The responses of this study were global yield, TAC, TPC and TFC.

### Calculation of global yield recovery

The global yield recovery was calculated using Eq. (1),

3 $$\mathrm{GY }\left(\mathrm{\%}\right)= \frac{{m}_{a}}{{m}_{ab}} \times 100,$$

where m_a_ is the mass of the extract (g) and m_ab_ is the mass of the sample (g).

### Extraction rate of bioactive compounds from roselle using ScCO_2_

Brunner’s and Esquivel's models were used to fit the extraction rate data of global yield. The Esquivel model has two adjustable parameters ($y$ and $k$) as shown in Eq. (4)^16^. The adjustable parameters are obtained from SFsolver Microsoft Excel 2019 (Microsoft^®^ Word 2019 MSO (Version 2212 Build 16.0.15928.20196) 64-bit).

4 $$\mathrm{GY }\left(\mathrm{mg}\right)=\mathrm{ y}\left(\frac{\mathrm{t}}{\mathrm{k}+\mathrm{t}}\right),$$

where $\mathrm{y}$ is the predicted global yield (mg), $\mathrm{k}$ are the adjustable parameters (s), and $\mathrm{t}$ is the extraction time (s). The extraction rate of the global yield can be obtained by $\mathrm{y}/\mathrm{k}$ (mg/s).

Brunner’s model also has two adjustable parameters (${Y}_{2}{ \mathrm{and} k}_{2}$) that represent a specific solution of Fick’s law, as shown in Eq. (5)^15^:

5 $$\mathrm{GY }\left(\mathrm{mg}\right)=\mathrm{ y}\left(1- {\mathrm{e}}^{-\mathrm{kt}}\right),$$

where $\mathrm{GY}$ is the global yield (mg), $\mathrm{y}$ is the predicted total phenolic or flavonoid content (mg/g), 1/k is the adjustable parameter and t is the extraction time (s). The extraction rate of global yield can be obtained by $\mathrm{y}/\mathrm{k}$ (mg/s).

### Single sphere model

A single sphere model was proposed by Reverchon^17^ with the following assumptions:

1. Intra particle of mass transfer is the main factor in the extraction process.
2. Resistance of mass transfer is zero between the extract and the solvent.
3. The roselle as a raw material is an inert porous sphere.
4. Particle size of roselle is homogenous.
5. Global yield is extracted move through the particles by process ‘similar to diffusion.

The diffusion equation for a constant diffusion coefficient takes the form of Eq. (6).

6 $${\mathrm{Y}}^{\mathrm{^{\prime}}}=\frac{{\mathrm{M}}_{\mathrm{t}}}{{\mathrm{M}}_{\infty }}=1-\frac{6}{{\uppi }^{2}}\sum_{\mathrm{n}-1}^{\infty }\frac{1}{{\mathrm{n}}^{2}}\mathrm{exp}\frac{{\mathrm{D}}_{\mathrm{e}}{\mathrm{tn}}^{2}{\uppi }^{2}}{{\mathrm{R}}^{2}},$$

where *Mt* is the total amount of diffusing substance at a specific time, *M∞* is the corresponding quantity after infinite time, *D*_*e*_ is the diffusivity coefficient (m^2^/s), R is the radius of the particle (m) and *t* is time (s). In this investigation, the solver in Microsoft Excel 2021 was used to determine the diffusivity coefficient. The adjustable parameters are obtained from SFsolver Microsoft Excel 2019 (Microsoft^®^ Word 2019 MSO (Version 2212 Build 16.0.15928.20196) 64-bit).

### Calculation of solubility

Determination of the solubility of global yield is according to Eq. (7)

7 $$S\left(\frac{g}{L}\right)= \frac{\mathrm{GY}\left(\mathrm{g}\right)}{{\mathrm{V}}_{co2}\left(\mathrm{L}\right)},$$

where $\mathrm{GY }(\mathrm{g})$ is the global yield (g) and ${\mathrm{\Delta V}}_{co2}(\mathrm{L})$ is the total CO_2_ consumption. The total CO_2_ consumption is measured based on the flowmeter of the CO_2_ pump.

### Average absolute relative deviation (AARD)

The AARD value is used to identify the optimal model between the model and the experimental data and is shown in Eq. (8).

8 $$AARD \left(\%\right)= \frac{100}{n} \sum_{i=1}^{n}\left|\frac{{E}_{model} - {E}_{exp}}{{E}_{exp}}\right|,$$

where n is the number of data points, ${E}_{model}$ is the model data, and ${E}_{exp}$ is the experimental data.

### Coefficient of determination (R^2^)

The R^2^ value is used to establish the optimal model between the model and experimental data, as shown by Eq. (9)

9 $${R}^{2}=1- \frac{\sum_{i}{\left({EM}_{i}-{ED}_{i}\right)}^{2}}{\sum_{i}{\left({x}_{i}-\overline{x }\right)}^{2}}.$$

Based on Eq. (9), $\sum_{i}{({EM}_{i}-{ED}_{i})}^{2}$ is the residual data (i.e. an error between the model and the experimental data). Meanwhile, $\sum_{i}{({x}_{i}-\overline{x })}^{2}$ is the variance of the data.

## Results and discussion

The dried roselle was grounded and sieved to various particle sizes of 250 µm < dp < 355 µm, 355 µm < dp < 425 µm and 425 µm < dp < 500 µm. The exclusion of particle size dp < 250 was due to the clogging of the apparatus. Fine particle of dried roselle will be compact in high pressure condition. Therefore, the extraction efficiency of the process will be reduced^20^. The moisture content of dried roselle was maintained below than 8%. This prevented the production of ice particles in the extraction tube^4^. Additionally, this development will block the ScCO_2_ extraction tube. In this research, the effect of particle size was examined and discussed with the responses of bioactive compound’s recovery, diffusivity, extraction rate and solubility.

### Effect of particle size on global yield, TAC, TPC and TFC

The particle size of the sample matrix is crucial for obtaining the maximum yield of supercritical carbon dioxide extraction using ethanol as an entrainer/cosolvent. Reducing the solute particle size will increase the extraction process yield^14^. Although reducing the particle size will increase the extract yield, previous researchers have reported that reducing the particle size does not always increase the yield extract. Therefore, preliminary studies are required to determine the optimal particle size to obtain the highest extract yield with various particle sizes^6^. In this investigation, roselle was produced via milling, where the milling process may enhance the specific area of the particle solute and damage the cell walls of roselle by decreasing the particle size^21^.

**Figure 2:**
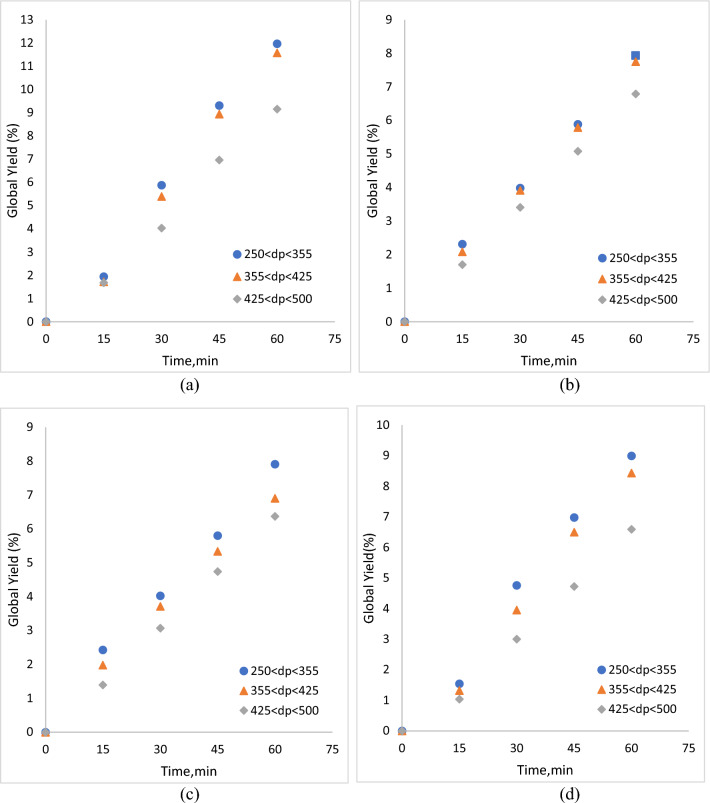
Influence of particle size on yield in extraction of roselle by ScCO_2_ at constant parameters (**a**) 10 MPa, 40 °C, (**b**) 10 MPa, 60 °C, (**c**) 30 MPa, 40 °C, and (**d**) 30 MPa, 60 °C.

**Figure 3:**
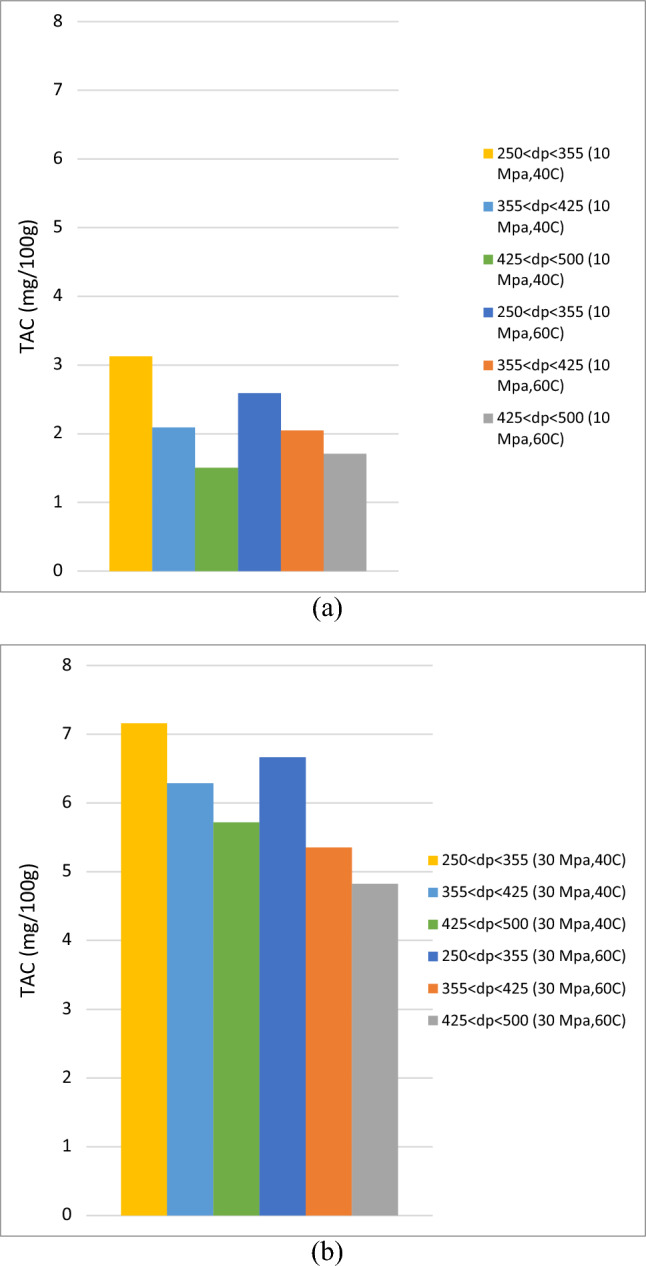
Influence of particle size on total anthocyanin compounds (TAC) in extraction of roselle by ScCO_2_ at constant pressure (**a**) 10 MPa and (**b**) 30 MPa.

**Figure 4:**
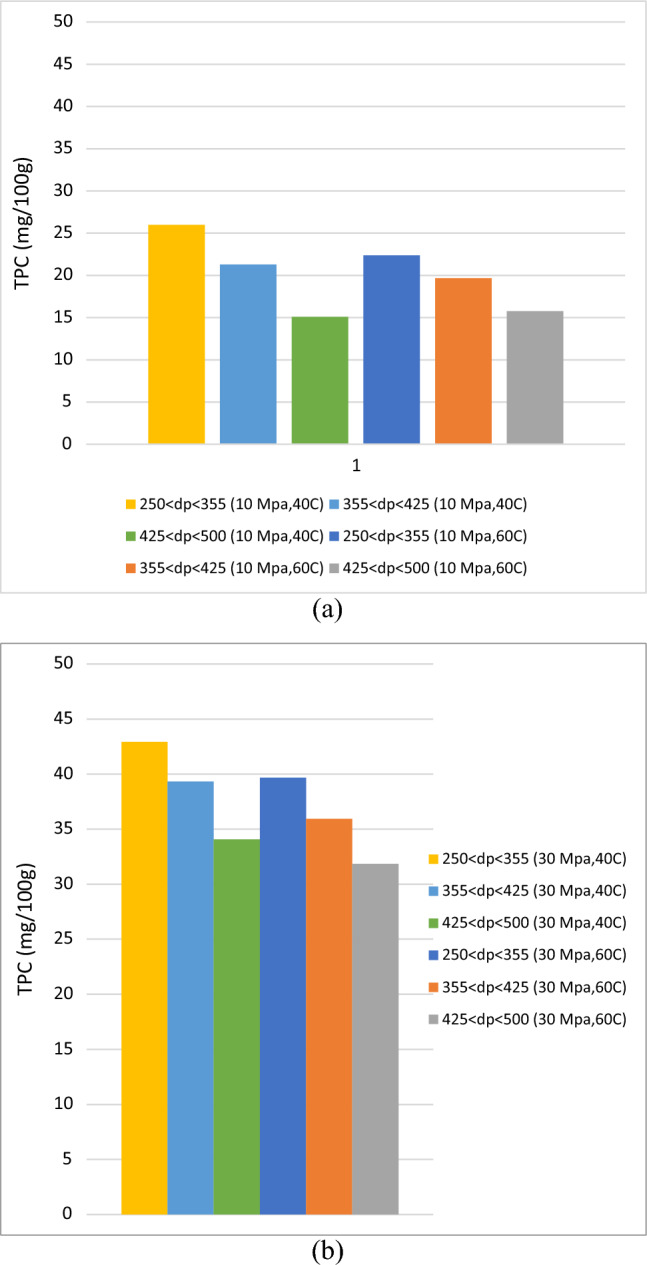
Influence of particle size on total phenolic compounds (TPC) in extraction of roselle by ScCO_2_ at constant pressure (**a**) 10 MPa and (**b**) 30 MPa.

**Figure 5:**
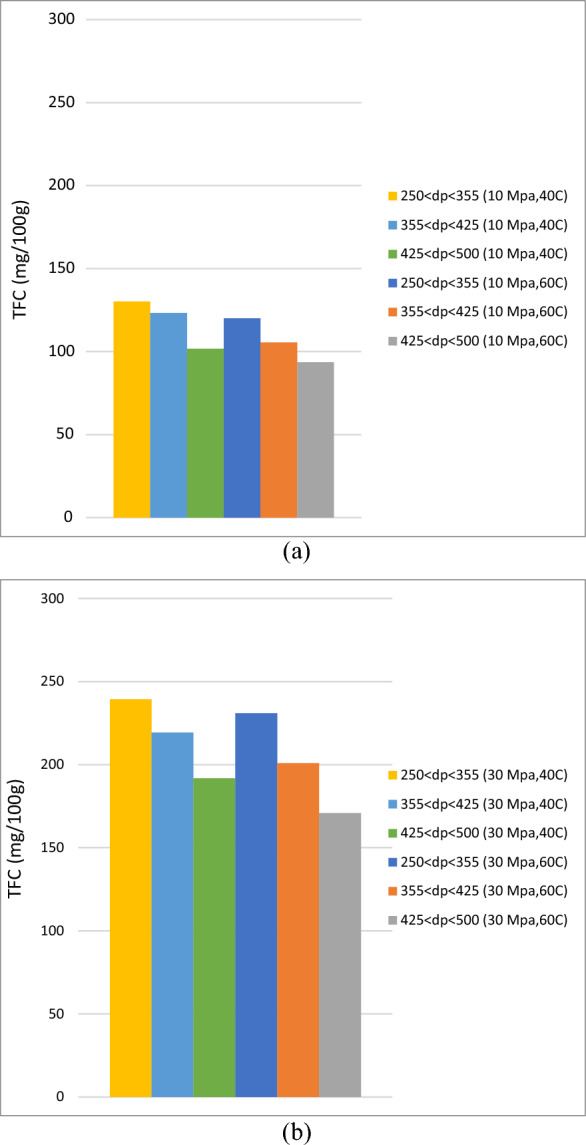
Influence of particle size on total flavonoid compounds (TFC) in extraction of roselle by ScCO_2_ at constant pressure (**a**) 10 MPa and (**b**) 10 MPa.

Figures 2, 3, 4 and 5 show the influence of particle size on global yield in the extraction of roselle by ScCO_2_ at constant parameters (a) 10 MPa, 40 °C, (b) 10 MPa, 60 °C, (c) 30 MPa, 40 °C, and (d) 30 MPa, 60 °C. Table 1 also shows the particle size parameters and responses of yield (%), TAC (mg/100 g), TPC (mg/100 g) and TFC (mg/100 g). The results show that decreasing the particle size from 425 µm < dp < 500 µm to 250 µm < dp < 355 µm at constant parameters (a) 10 MPa, 40 °C, (b) 10 MPa, 60 °C, (c) 30 MPa, 40 °C, and (d) 30 MPa, 60 °C increases the global yield, total anthocyanin content (TAC), total phenolic compounds (TPC) and total flavonoid compounds (TFC), as shown in Figs. 2, 3, 4 and 5. Reducing the particle size improves the sample contact area with extraction solvents. Grinding produced coarser and smaller samples, but powdering produced more homogeneous and smaller particles, resulting in improved surface contact with extraction solvents. The solvent must make contact with the solute, and a particle size less than 0.5 mm is optimal for efficient extraction. Reverchon et al.^22^ also mentioned that larger particles may result in lengthy diffusion-controlled solvent extraction and that slow diffusion can significantly impact the extraction kinetics.

Sodeifian et al.^23^ discovered that particle size may have two contradictory impacts on extraction yield. In contrast, the grinding procedure increases the contact area between the supercritical fluid and the samples, hence increasing the extraction yield. In fact, from a mass transfer stand point, additional particle size reduction may inhibit the extraction process, since the volatile oil may be simply reabsorbed on matrix surfaces, resulting in a decrease in solute transport.

In addition, the small size of particles may contribute to bed caking formation (particles sticking together, channelling) along the bed through which ScCO_2_ can flow more efficiently, resulting in insufficient contact between the sample and ScCO_2_ and, ultimately, insufficient extraction yield. Furthermore, Putra et al.^6^ also found that the fine particle of peanut skin (dp < 250 µm) give low extraction efficiency of catechin as polar compounds. The polarity of catechin is similar to the anthocyanin, thus the process can be compared. This phenomenon is due to the small size of particles may contribute to bed caking formation during the extraction. It reduced the extraction efficiency of ScCO_2_. It is also reducing the diffusivity of CO_2_ to break the cell wall of materials.

Additionally, Darbandi et al.^24^ discovered that grinding the material prior to the extraction procedure enhances the interfacial area. In addition, crushing the particles facilitates the release of oil from the cells. During the grinding process, certain cells are damaged, and the contained oil is released from the shattered cells. ScCO_2_ makes this quantity of released extract containing bioactive compounds more accessible, and it dissolves rapidly in the solvent. Milling the particles reduces the intraparticle barrier to mass transfer in addition to these benefits. Consequently, the route of diffusion in solids becomes shorter. Thus, the solute will be easier to move, and the extraction yield will increase. The aforementioned causes may explain why the extraction yield increases as the particle size decreases.

Sabio et al.^25^ also studied the impact of particle size on the extraction yield of a combination of tomato skin and seeds. Contrary to this research, greater particle size increases yield more than smaller particle size at constant pressure of 300 bar, temperature of 60 °C, and flow rate of 0.792 kg/h for two distinct particle sizes. Larger particles in this instance give a unique diffusion-controlled extraction but need a lengthy processing time. The extraction yield increased as the particle size decreased. This is the result of the increased interfacial area.

### Influence of the particle size of roselle on the extraction rate of global yield

**Figure 6:**
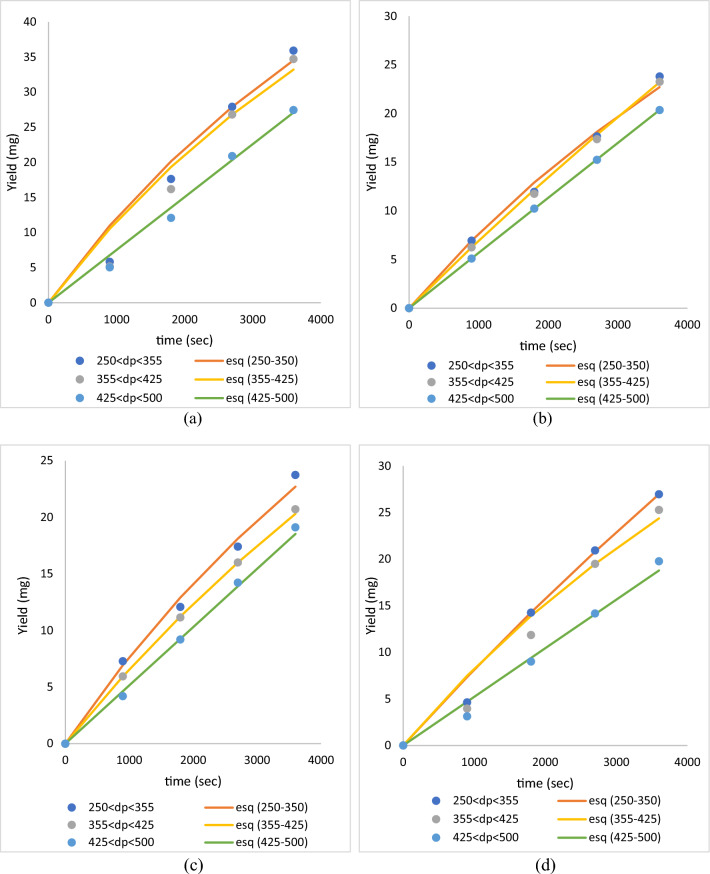
The experimental data with different particle size is fitted by Esquivel data at constant parameters (**a**) 10 MPa, 40 °C (**b**) 10 MPa 60 °C (**c**) 30 MPa 40 °C and (**d**) 30 MPa 60 °C.

Brunner's and Esquivel's models were used for the investigation of the extraction rate because they require fewer adjustable parameters and are simply fitted to experimental data^9^. Table 2 demonstrates that the findings of Brunner and Esquivel's model fit the global yield recovery from roselle using ScCO_2_. Meanwhile, Fig. 6 shows that the experimental data are fitted by Esquivel’s model at pressures of 10 and 30 MPa, temperatures of 40 and 60 °C, and particle sizes (250 µm < dp < 355 µm, 355 µm < dp < 425 µm and 425 µm < dp < 500 µm).

**Table 2:** The experimental data fitted by Esquivel and Brunner models.

Run	Temp, °C	P, Mpa	F_CO2_, ml/min	Dp, µm	Global Yield, %	Esquivel						Brunner			
						k	y	k/y (mg/s)	%AARD	R^2^	k	1/y	k/y (mg/s)	%AARD	R^2^
1	40	10	4	250 < dp < 355	7.91	1.13E + 04	9.41E + 01	8.32E−03	3.00	1.00	1.88E−04	4.46E + 01	8.37E−03	3.15	1.00
2				355 < dp < 425	6.90	3.46E + 04	2.46E + 02	7.12E−03	1.26	1.00	1.53E−04	4.87E + 01	7.47E−03	3.67	0.99
3				425 < dp < 500	6.37	6.16E + 05	3.50E + 03	5.69E−03	0.18	1.00	1.18E−04	5.35E + 01	6.31E−03	4.01	1.00
4	60	10	4	250 < dp < 355	7.94	8.96E + 03	1.21E + 02	1.35E−02	12.70	0.99	8.90E−05	1.31E + 02	1.16E−02	10.23	0.99
5				355 < dp < 425	7.75	9.12E + 03	1.17E + 02	1.29E−02	14.40	0.98	9.75E−05	1.16E + 02	1.13E−02	12.30	0.99
6				425 < dp < 500	6.79	1.11E + 10	8.36E + 07	7.53E−03	8.08	1.00	6.63E−05	1.27E + 02	8.45E−03	9.76	0.99
7	40	30	4	250 < dp < 355	11.97	1.13E + 04	9.41E + 01	8.32E−03	3.99	1.00	1.88E−04	4.46E + 01	8.37E−03	4.15	1.00
8				355 < dp < 425	11.57	1.50E + 04	1.05E + 02	7.00E−03	0.56	1.00	1.18E−04	5.92E + 01	6.96E−03	0.63	1.00
9				425 < dp < 500	9.15	3.65E + 10	1.88E + 08	5.15E−03	3.13	1.00	3.94E−05	1.41E + 02	5.55E−03	4.35	1.00
10	60	30	4	250 < dp < 355	8.99	2.91E + 04	2.45E + 02	8.42E−03	7.53	0.99	7.11E−05	1.19E + 02	8.45E−03	7.72	0.99
11				355 < dp < 425	8.43	1.09E + 04	9.83E + 01	9.00E−03	13.15	0.99	1.94E−04	4.78E + 01	9.28E−03	13.91	0.98
12				425 < dp < 500	6.59	1.97E + 08	1.03E + 06	5.22E−03	8.70	1.00	1.18E−04	5.20E + 01	6.13E−03	11.95	0.99
Average					8.36	3.99E + 09	2.27E + 07	0.01	0.99	6.39	0.00	82.00	0.01	7.15	0.99

On the basis of the average coefficients of determination (R^2^) and the average absolute relative deviation (AARD), the most appropriate model was identified and is displayed in Table 2. The mathematical model with the greatest average coefficients of determination (i.e., R^2^ > 1) and the lowest average absolute relative deviation (AARD < 10%) was the most suitable. Table 2 demonstrates that Esquivel's model provided a better fitting for global yield recovery than Brunner's model, indicating that it might give more accurate data on the extraction rate of global yield.

**Figure 7:**
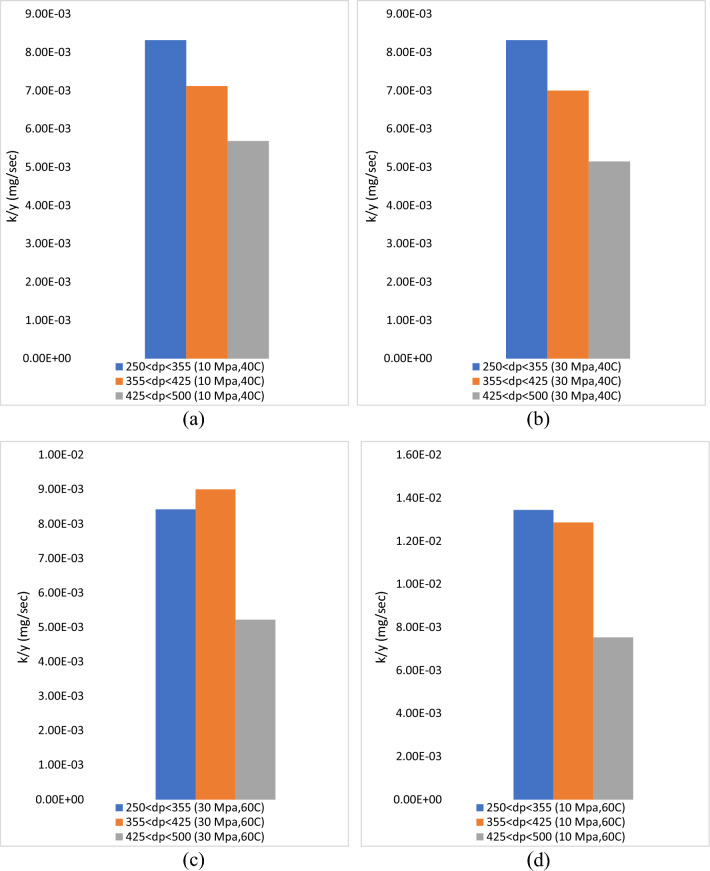
The extraction rate of global yield obtained from Esquivel model with different particle size is fitted by Esquivel data at constant parameters (**a**) 10 MPa, 60 °C (**b**) 10 MPa 40 °C (**c**) 30 MPa 40 °C and (**d**) 30 MPa 60 °C.

The average extraction rate of the global yield was 0.01 mg/s, as shown in Table 2. On the other hand, a pressure of 10 MPa, temperature of 60 °C and particle size of 250 µm < dp < 355 µm gave the maximum extraction rate (1.35 E−02 mg/s) and at pressure 30 MPa, tempereature 40 °C and particle size 425 µm < dp < 500 µm gives the minimum extraction rate (5.15E−03 mg/s). Overall, the particle size of 250 µm < dp < 355 µm gives a higher extraction rate of global yield compared to 355 µm < dp < 425 µm and 425 µm < dp < 500 µm, as shown in Fig. 7. It is often assumed that cavitation effects, heat impacts, and mechanical effects have a substantial impact on the ScCO_2_ extraction method^12^. These actions result in cell wall disintegration, particle size reduction, and an increase in reaction rate by mass transfer of the cell wall, without altering the extracts' structure or function^26,27^. Therefore, a smaller particle size might improve the particle cell wall damage, hence increasing the extraction rate and global yield.

### Influence of the particle size of roselle on diffusivity of global yield

**Figure 8:**
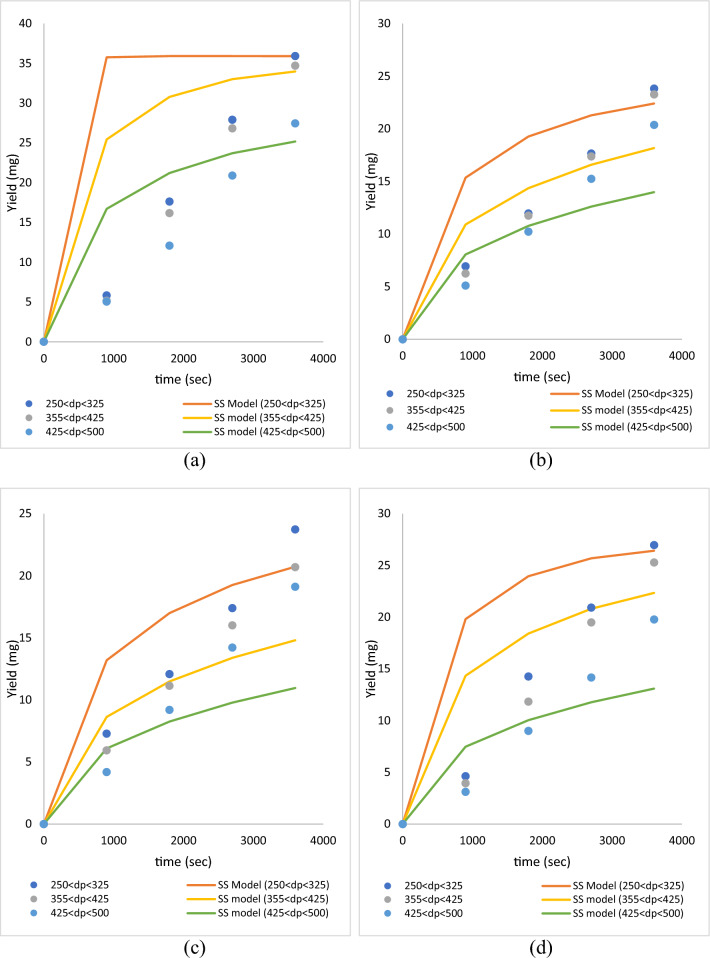
The experimental data with different particle size is fitted by single sphere model data at constant parameters (**a**) 10 MPa, 40 °C (**b**) 10 MPa 60 °C (**c**) 30 MPa 40 °C and (**d**) 30 MPa 60 °C.

To determine the diffusivity coefficient and mass transfer between solvent and solute on supercritical carbon dioxide, the single sphere model is typically employed as the kinetic model. The single sphere model is easier to use than other kinetic models because it has a single adjustable parameter compared to other models^28^. Table 3 demonstrates that the findings of the single sphere model fit the global yield recovery from roselle using ScCO_2_. Meanwhile, Fig. 8 shows that the experimental data are fitted by a single sphere model at pressures of 10 and 30 MPa, temperatures of 40 and 60 °C, and particle sizes (250 µm < dp < 355 µm, 355 µm < dp < 425 µm and 425 µm < dp < 500 µm). On this variable, the single sphere model is slightly successful in fitting the experimental data with a higher percentage of error. This is because the single sphere model is suitable for high pressure conditions; hence, this pressure is not enough to obtain a high diffusivity of solvent^29^. Table 3 shows that the design of the experiment for this study based on all single sphere model experiments has an error higher than 5%.

**Table 3:** The experimental data fitted by single sphere models.

Run	Temp, °C	P, Mpa	F_CO2_, ml/min	F_Modifier_, ml/min	Time, min	Dp, µm	Global yield, %	De, mg/s^2^	%AARD
1	40	10	4	0.24	60	250 < dp < 355	7.91	4.27E−12	29.06
2						355 < dp < 425	6.91	3.67E−12	22.88
3						425 < dp < 500	6.37	3.54E−12	23.17
4	60	10	4	0.24	60	250 < dp < 355	7.93	3.72E−11	42.35
5						355 < dp < 425	7.75	1.20E−11	40.28
6						425 < dp < 500	6.78	1.02E−11	31.70
7	40	30	4	0.24	60	250 < dp < 355	11.96	2.88E−12	24.55
8						355 < dp < 425	1157	2.78E−12	20.32
9						425 < dp < 500	9.15	2.17E−12	28.00
10	60	30	4	0.24	60	250 < dp < 355	8.99	6.22E−12	32.35
11						355 < dp < 425	8.43	5.88E−12	30.79
12						425 < dp < 500	6.59	3.19E−12	29.08
Average							8.36	7.83E−12	29.54

**Figure 9:**
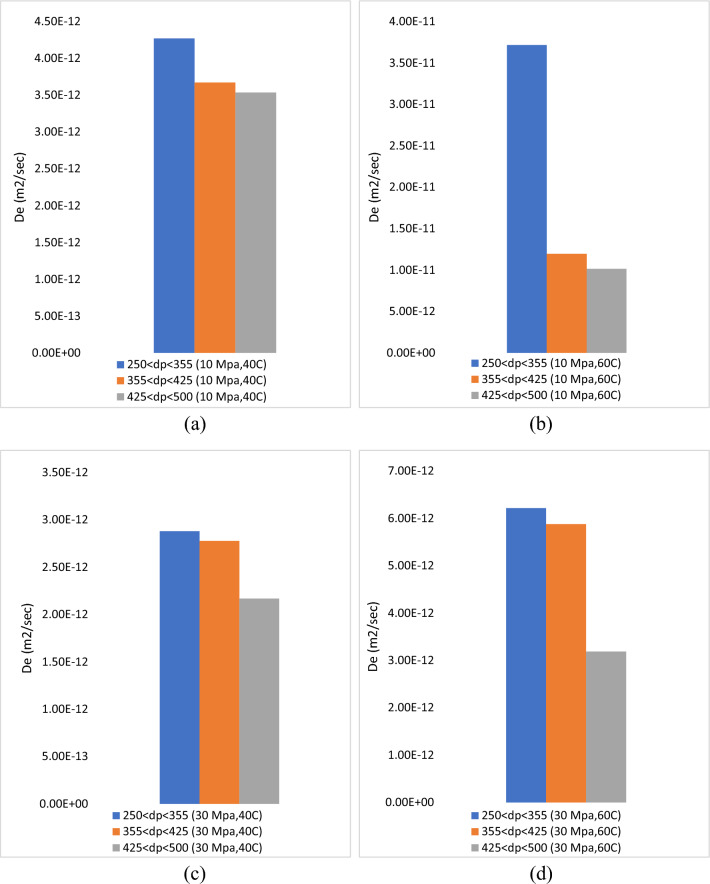
The diffusivity coefficient of global yield obtained from single sphere model with different particle size is fitted by Esquivel data at constant parameters (**a**) 10 MPa, 40 °C (**b**) 10 MPa 60 °C (**c**) 30 MPa 40 °C and (**d**) 30 MPa 60 °C.

Table 3 shows that the average diffusivity coefficient was 7.83E−12 m^2^/s. on the other hand, the pressure 10 MPa, tempereature 60 °C and particle size 250 µm < dp < 355 µm gives the maximum diffusivity coefficient (3.72E−11 m^2^/s) and at pressure 30 MPa, tempereature 40 °C and particle size 425 µm < dp < 500 µm gives the minimum diffusivity coefficient (2.17E−12 m^2^/s). Moreover, the results show that a particle size of 250 µm < dp < 355 µm at different lowest and highest constant pressures and temperatures gives the maximum oil yield and diffusivity coefficient. This confirms that 250 µm < dp < 355 µm is the optimum particle size due to the high diffusivity coefficient of the extraction process, as shown in Fig. 9.

The high diffusivity coefficient indicates the high mass transfer process between supercritical carbon dioxide and extract as a solute. Therefore, increasing the diffusivity of the extraction will enhance the kinetic transfer of the extract to dissolve into the solvent^30^. Decreasing De indicates the difficulty of supercritical solvent diffusion into the pores of particles to dissolve the solute and implies that the mass transfer resistance is mainly located in the solid phase^31^.

### Influence of the particle size of roselle on the solubility of global yield, TAC, TPC and TFC

Solubility data are a valuable measure, especially in ScCO_2_ extraction, to encourage the extraction process. Therefore, the extraction conditions can be determined appropriately by using this information. The solubility of roselle extracts in ScCO_2_ under various operating conditions is shown in Table 4, where the average solubility of global yield, TAC, TPC and TFC were 1.05 g/L, 0.17 mg/L, 1.19 mg/L and 6.69 mg/L, respectively. The solubility was evaluated at pressures of 10 and 30 MPa, temperatures of 40 and 60 °C and particle sizes of 250 µm < dp < 355 µm, 355 µm < dp < 425 µm and 425 µm < dp < 500 µm. The value of ScCO2 density was obtained from Engineering toolbox (https://www.engineeringtoolbox.com/carbon-dioxide-density-specific-weight-temperature-pressure-d_2018.html).

**Table 4:** Particle size effect on solubility of roselle extract and its bioactive compounds.

Run	Temp, °C	P, Mpa	F_CO2_, ml/min	F_Modifier_, ml/min	Time, min	Dp, µm	Global yield, %	Solubility global yield, g/L	Solubility TAC, mg/L	Solubility TPC, mg/L	Solubility TFC, mg/L
1	40	10	4	0.24	60	250 < dp < 355	7.91	0.99	0.13	1.08	5.43
2						355 < dp < 425	6.91	0.86	0.09	0.89	5.14
3						425 < dp < 500	6.37	0.80	0.06	0.63	4.24
4	60	10	4	0.24	60	250 < dp < 355	7.93	0.99	0.11	0.93	5.00
5						355 < dp < 425	7.75	0.97	0.09	0.82	4.39
6						425 < dp < 500	6.78	0.85	0.07	0.66	3.90
7	40	30	4	0.24	60	250 < dp < 355	11.96	1.50	0.30	1.79	9.97
8						355 < dp < 425	1157	1.45	0.26	1.64	9.14
9						425 < dp < 500	9.15	1.14	0.24	1.42	7.99
10	60	30	4	0.24	60	250 < dp < 355	8.99	1.12	0.28	1.65	9.63
11						355 < dp < 425	8.43	1.05	0.22	1.50	8.37
12						425 < dp < 500	6.59	0.82	0.20	1.33	7.12
Average							8.36	1.05	0.17	1.19	6.69

**Figure 10:**
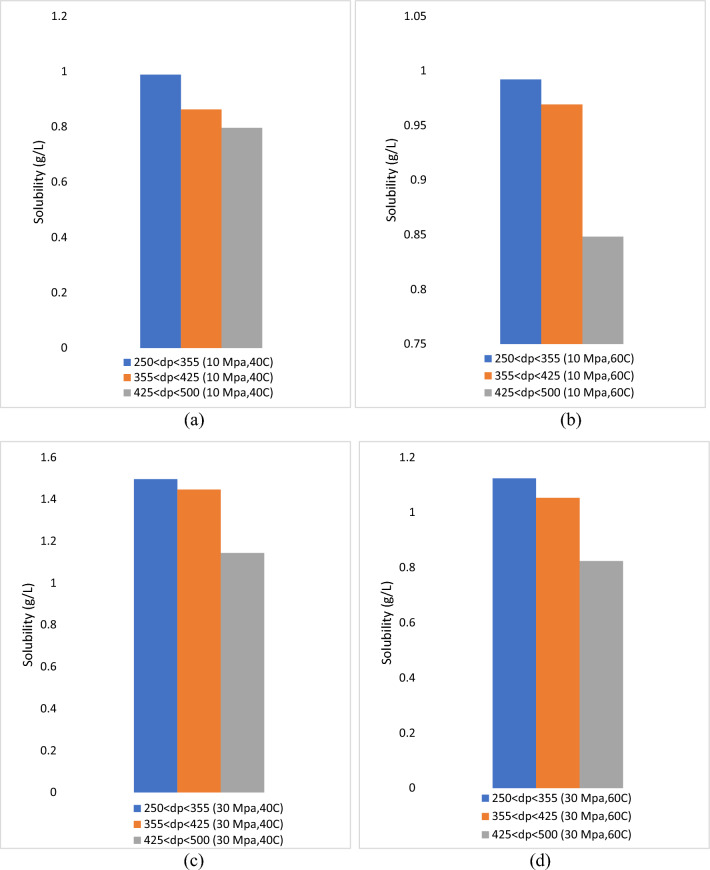
The solubility of global yield with different particle size at constant parameters (**a**) 10 MPa, 40 °C (**b**) 10 MPa 60 °C (**c**) 30 MPa 40 °C and (**d**) 30 MPa 60 °C.

Figure 10 shows that the higher pressures and lowest particle size increased the solubility of the extract because the increase in pressure enhances the density of solvents. In addition, the increase in pressure increases the solvation power of the ScCO_2_ mixture to roselle^32^. Table 4 also shows that the lowest particle size of 250 µm < dp < 355 µm gives the highest solubility TAC, TPC and TFC. Fine particles are simpler to extract because they have a large surface area per unit volume, contain a high proportion of “free oil” and need less distance for the “tied oil” to reach the surface, which minimizes the internal mass transfer resistance^33,34^. Reverchon^17^ found that the influence of particle size on cumulative solubility may be described in terms of mass transfer resistances. The extraction of solute from the solid matrix presents two forms of mass transfer resistances, namely, internal mass transfer resistance and exterior mass transfer resistance^35^. If the internal mass transfer mechanisms comprise the governing phase of the extraction process, the particle size of the roselle matter may greatly impact the cumulative extraction yield. In this condition, extraction from various particle sizes will mostly depend on the length of the diffusion path^20^. If internal mass transfer or equilibrium is the controlling stage of the process, the particle size could significantly impact the extraction rate/solubility^36^.

## Conclusion

Roselle (*Hibiscus sabdariffa*) provides anthocyanin, phenolic, flavonoid, and antioxidant components, among other things. The essential compounds in roselle are extracted using ScCO_2_ as a green solvent. Mechanical parameters, such as the particle size of materials, have been slightly studied by researchers to enhance the efficiency of ScCO_2_ extraction. The particle size of roselle was studied to obtain the best particle size with high global yield and anthocyanin, phenolic and flavonoid compounds. The greatest recovery was 11.96% yield, 7.16 mg/100 g TAC, 42.93 mg/100 g TPC and 239.36 mg/100 g TFC under the conditions of 30 MPA, 40 °C and 250 µm < dp < 355 µm, respectively. The extraction rate of ScCO_2_ in roselle extraction was ranged from 5.19 E−03 to 1.35 E−03 mg/s fitted using the Esquivel model. The diffusivity coefficient of ScCO_2_ ranged from 2.17E−12 to 3.72E−11 mg/s^2^, as fitted by a single sphere model. The maximum solubility of global yield, TAC, TPC and TFC in ScCO_2_ was 1.50 g/L, 0.3 mg/L, 1.69 mg/L and 9.97 mg/L, respectively, with a particle size of 250 µm < dp < 355 µm. In conclusion, a reduced particle size enhances the solubility, diffusivity, extraction rate and recovery of the extract.

## Data Availability

The data that support the findings of this study are available from the corresponding author upon reasonable request.

## References

[1] Idham Z (2022). Improvement of extraction and stability of anthocyanins, the natural red pigment from roselle calyces using supercritical carbon dioxide extraction. J. CO2 Util..

[2] Rizkiyah DN (2022). Optimization of red pigment anthocyanin recovery from *Hibiscus sabdariffa* by subcritical water extraction. Processes.

[3] Rizkiyah DN, Jusoh WMSW, Idham Z, Putra NR, Che Yunus MA (2022). Investigation of phenolic, flavonoid and antioxidant recovery and solubility from roselle using supercritical carbon dioxide: Experimental and modelling. J. Food Process. Preserv..

[4] Daud NM (2022). Valorisation of plant seed as natural bioactive compounds by various extraction methods: A review. Trends Food Sci. Technol..

[5] Putra, N. R. *et al.* A new solubility model for competing effects of three solvents: Water, ethanol, and supercritical carbon dioxide. *Sep. Sci. Technol.* 1–7 (2022).

[6] Putra NR (2018). Effect of particle size on yield extract and antioxidant activity of peanut skin using modified supercritical carbon dioxide and soxhlet extraction. J. Food Process. Preserv..

[7] Sodeifian G, Ardestani NS, Sajadian SA, Ghorbandoost S (2016). Application of supercritical carbon dioxide to extract essential oil from *Cleome coluteoides* Boiss: Experimental, response surface and grey wolf optimization methodology. J. Supercrit. Fluids.

[8] Sodeifian G, Sajadian SA, Saadati Ardestani N (2016). Optimization of essential oil extraction from *Launaea acanthodes* Boiss: Utilization of supercritical carbon dioxide and cosolvent. J. Supercrit. Fluids.

[9] Putra NR (2022). Extraction rate of valuable compounds from peanut skin waste by ethanol-assisted supercritical carbon dioxide: Modelling and optimization. Malays. J. Fundam. Appl. Sci..

[10] Sodeifian G, Sajadian SA, Saadati Ardestani N (2017). Supercritical fluid extraction of omega-3 from *Dracocephalum kotschyi* seed oil: Process optimization and oil properties. J. Supercrit. Fluids.

[11] Abdul Aziz AH (2022). Solubility of rosmarinic acid in supercritical carbon dioxide extraction from *Orthosiphon stamineus* leaves. ChemEngineering.

[12] Lu Y (2019). Relationship between pore structure and mechanical properties of shale on supercritical carbon dioxide saturation. Energy.

[13] Sodeifian G, Sajadian SA, Ardestani NS (2016). Optimization of essential oil extraction from *Launaea acanthodes* Boiss: Utilization of supercritical carbon dioxide and cosolvent. J. Supercrit. Fluids.

[14] Yiana LN (2021). Supercritical carbon dioxide extraction of hevea brasiliensis seeds: Influence of particle size on to oil seed recovery and its kinetic. Malays. J. Fundam. Appl. Sci..

[15] Brunner, G. *Gas Extraction: An Introduction to Fundamentals of Supercritical Fluids and the Application to Separation Processes*. Vol. 4 (Springer Science & Business Media, 2013).

[16] Esquıvel M, Bernardo-Gil M, King M (1999). Mathematical models for supercritical extraction of olive husk oil. J. Supercrit. Fluids.

[17] Reverchon E (1997). Supercritical fluid extraction and fractionation of essential oils and related products. J. Supercrit. Fluids.

[18] Lee J, Durst RW, Wrolstad RE, Kupina C (2005). Determination of total monomeric anthocyanin pigment content of fruit juices, beverages, natural colorants, and wines by the pH differential method: collaborative study. J. AOAC Int..

[19] Putra NR, Rizkiyah DN, Machmudah S, Shalleh LM, Che Yunus MA (2020). Recovery and solubility of flavonoid and phenolic contents from Arachis Hypogea in supercritical carbon dioxide assisted by ethanol as cosolvent. J. Food Process. Preserv..

[20] Cissé M (2012). Aqueous extraction of anthocyanins from *Hibiscus sabdariffa*: Experimental kinetics and modeling. J. Food Eng..

[21] Mohd-Nasir H (2021). Optimization of the supercritical carbon dioxide extraction of Quercus infectoria galls extracts and its bioactivities. J. Food Process. Preserv..

[22] Reverchon KA, Marrone C (2000). Supercritical CO_2_ extraction of hiprose seed oil: Experiments and mathematical modelling. Chem. Eng. Sci..

[23] Sodeifian G, Sajadian SA, Honarvar B (2018). Mathematical modelling for extraction of oil from *Dracocephalum kotschyi* seeds in supercritical carbon dioxide. Nat. Prod. Res..

[24] Darbandi T, Honarvar B, Sinaei Nobandegani M, Rezaei A (2013). Extraction of Ziziphora tenuior essential oil using supercritical CO_2_. Eur. J. Exp. Biol..

[25] Sabio E (2003). Lycopene and β-carotene extraction from tomato processing waste using supercritical CO_2_. Ind. Eng. Chem. Res..

[26] Toma M, Vinatoru M, Paniwnyk L, Mason TJ (2001). Investigation of the effects of ultrasound on vegetal tissues during solvent extraction. Ultrason. Sonochem..

[27] Wen C (2018). Advances in ultrasound assisted extraction of bioactive compounds from cash crops—A review. Ultrason. Sonochem..

[28] Roy BC, Goto M, Hirose T (1996). Extraction of ginger oil with supercritical carbon dioxide: experiments and modeling. Ind. Eng. Chem. Res..

[29] Abdul Aziz AH, Putra NR, Kong H, Che Yunus MA (2020). Supercritical carbon dioxide extraction of sinensetin, isosinensetin, and rosmarinic acid from *Orthosiphon stamineus* leaves: Optimization and modeling. Arab. J. Sci. Eng..

[30] Putra, N. R., Idham, Z. B., Machmudah, S., Ruslan, M. S. H. b. & Che Yunus, M. A. Extraction of peanut skin oil by modified supercritical carbon dioxide: Empirical modelling and optimization. *Sep. Sci. Technol.* 1–9 (2018).

[31] Nasir HM, Salleh LM, Ruslan MSH, Zahari MAM (2017). Single sphere model fitting of supercritical carbon dioxide extraction from *Quercus infectoria* galls. Malays. J. Fundam. Appl. Sci..

[32] Quispe-Fuentes I, Uribe E, López J, Contreras D, Poblete J (2022). A study of dried mandarin (*Clementina orogrande*) peel applying supercritical carbon dioxide using co-solvent: Influence on oil extraction, phenolic compounds, and antioxidant activity. J. Food Process. Preserv..

[33] Duba KS, Fiori L (2015). Supercritical CO_2_ extraction of grape seed oil: Effect of process parameters on the extraction kinetics. J. Supercrit. Fluids.

[34] Sovová H (1994). Rate of the vegetable oil extraction with supercritical CO_2_—I. Modelling of extraction curves. Chem. Eng. Sci..

[35] Mens-Appamana W (2022). Investigation of mass transfer and hydrodynamics of liquid–liquid extraction in spinning disc reactor by computational fluid dynamics simulation. Results Eng..

[36] Zabihi S, Esmaeili-Faraj SH, Borousan F, Hezave AZ, Shirazian S (2020). Loxoprofen solubility in supercritical carbon dioxide: Experimental and modeling approaches. J. Chem. Eng. Data.

